# A review of the current state of single-cell proteomics and future perspective

**DOI:** 10.1007/s00216-023-04759-8

**Published:** 2023-06-07

**Authors:** Rushdy Ahmad, Bogdan Budnik

**Affiliations:** Wyss Institute for Biologically Inspired Engineering at Harvard University, 3 Blackfan Circle, Boston, MA 02115 USA

**Keywords:** Single-cell proteomics, SCP, Review, Current state, Future perspective

## Abstract

Single-cell methodologies and technologies have started a revolution in biology which until recently has primarily been limited to deep sequencing and imaging modalities. With the advent and subsequent torrid development of single-cell proteomics over the last 5 years, despite the fact that proteins cannot be amplified like transcripts, it has now become abundantly clear that it is a worthy complement to single-cell transcriptomics. In this review, we engage in an assessment of the current state of the art of single-cell proteomics including workflow, sample preparation techniques, instrumentation, and biological applications. We investigate the challenges associated with working with very small sample volumes and the acute need for robust statistical methods for data interpretation. We delve into what we believe is a promising future for biological research at single-cell resolution and highlight some of the exciting discoveries that already have been made using single-cell proteomics, including the identification of rare cell types, characterization of cellular heterogeneity, and investigation of signaling pathways and disease mechanisms. Finally, we acknowledge that there are a number of outstanding and pressing problems that the scientific community vested in advancing this technology needs to resolve. Of prime importance is the need to set standards so that this technology becomes widely accessible allowing novel discoveries to be easily verifiable. We conclude with a plea to solve these problems rapidly so that single-cell proteomics can be part of a robust, high-throughput, and scalable single-cell multi-omics platform that can be ubiquitously applied to elucidating deep biological insights into the diagnosis and treatment of all diseases that afflict us.

## Introduction

Advances in single-cell RNA sequencing (scRNA-seq) and genomics analysis have made it abundantly clear that observed biological variability can be ascribed directly to individual cells instead of being averaged over bulk or complex tissue [1]. Deep transcriptomic analysis over the last 10 years has revealed the existence of cellular complexity and heterogeneity which confounds our ability to deliver actionable results that can ultimately be employed by clinicians to help patients. The analysis of complex samples, such as plasma, cerebrospinal fluid (CSF), and model organoids, by existing bulk analytical methods have, therefore, failed to reveal the deep insights needed to develop impactful therapeutic or diagnostic interventions. Although scRNA-seq has ushered in the era of single-cell biology, it does not tell the whole picture, since the functional actors of cells are proteins, and we need access to the proteome at single-cell resolution for a holistic cellular picture. Given that proteins are the primary drivers of cellular function, a deep characterization of the proteome of individual cells by mass spectrometry and other proteomics techniques offers the research community an extraordinary and complimentary view of the cellular landscape.

Single-cell proteomics (SCP) is now at an inflection point in its march toward truly revolutionizing the field of single-cell biology. We know that observed proteomic differences poorly correlate with corresponding transcriptomic differences between biological states [2]. A recent study comparing single-cell RNA and proteome levels showed that the proteome is stable while the transcriptome is more variable, signifying regulation of translation and inviting its exploration at the single-cell level [3]. This makes it clear that in order to form an accurate and deeper understanding of translational regulation, especially given cellular heterogeneity in any given disease, SCP is a necessary and complimentary tool to scRNA-seq. Cellular diversity and heterogeneity cannot be addressed by bulk proteomics techniques, since it queries protein expression of many thousands of cells and provides an average protein expression profile which in most cases obscures crucial differences between individual cells. Being able to observe cell-to-cell differences is critical to accurately understanding the biology of disease. Also, in order to study protein expression changes in a dynamic manner in living cells, we can only employ SCP since bulk proteomics uses cell aggregates that have already been fixed and processed. Furthermore, the study of rare cell populations is not accessible to bulk proteomics since it does not have the sensitivity to detect such rare cells, like cancer stem cells. The role of rare cells in disease can only be addressed by SCP techniques.

Now, SCP not only gives us high-resolution data at the proteome level but also provides insight into post-translational modifications (PTMs), which is absolutely inaccessible to transcriptomic analysis. This type of single-cell-resolution proteomics data is necessary to study early cell-signaling events and changes due to cellular environmental fluctuations from drug intervention or disease influences. Even though the proteomics field has been using bulk proteomics techniques to address questions in cell-signaling dynamics, the results from such an analysis cannot provide accurate answers since it averages the signal coming from a heterogenous population of cells. With SCP, we can now ask key biological questions, such as signaling mechanisms based on protein binding, modifications, and degradation, which have long remained hidden from explorations using bulk proteomics techniques. We know that the abundance and activity of many proteins are regulated by degradation and post-translational modifications, and these cannot be inferred from genomic and transcriptomic measurements. Additionally, sequencing at the transcriptomic and genomic level does not shed any light on protein–protein interactions and protein localization, which are critical to understand numerous signaling pathways [4].

The research community clearly understands the need for single-cell protein measurements, and this has invigorated the development of single-cell mass spectrometry (MS) methods that can identify and quantify thousands of proteins from single cells at an unprecedented scale [5–8]. All these single-cell MS technologies aim to provide efficient delivery of peptides from single cells to the MS instruments via innovative sample preparation methods [9, 10]. At this juncture, any laboratory that employs quantitative MS proteomics techniques should be able to carry out quantitative protein analysis at single-cell resolution. But achieving such a goal requires robust SCP methods and well-defined uniform standards that can allow researchers to perform experiments in a high-throughput automated manner, at scale, and on widely available equipment and integrated data analysis platforms. A recent 2023 publication by Gatto et al. provides [11] “recommendations for performing, benchmarking, and reporting single-cell proteomics experiments.” In this timely report, the authors propose an initial set of guidelines, standardized metrics, best practices, quality controls, and recommendations for reporting single-cell proteomics data. Furthermore, a timely 2023 review article,^12^ by Vanderaa and Gatto, provides the current state of single-cell proteomics data analysis. In this article, the authors express the need for benchmarking computational workflows and standardizing computational tools and data. Now, although there have been significant advances in SCP methodologies, we are still far behind in the development of high-throughput, automated, robust, and scalable technologies for SCP analysis. We strongly believe that the development in these areas will empower the research community to generate reproducible and actionable SCP data at large scale by comprehensively exploring biology at single-cell resolution.

It is clear that single-cell omics technologies can aid in examining mRNA, DNA, and proteins in cells, but for a true holistic picture of a cell in a given context, we need to also understand the chemical processes involving metabolites in cells. Additionally, a survey of metabolite levels^13^ or “metabolomics” can provide a molecular readout that reflects the physiological state of a given cell and this information also needs to be folded into the single-cell biology landscape. To deepen the proteome coverage, reduce missing values, and significantly improve throughput in order to make adequately powered clinical research possible are goals that the entire SCP community shares [14–16]. To achieve these goals, the SCP community must overcome significant but not insurmountable challenges. Nevertheless, the future is bright for single-cell biology research, but the different single-cell omics technologies will ultimately need to come together to provide a truly holistic and comprehensive view of single-cell biology. With this future in mind, we provide the following review of the current state of SCP, focusing on the most popular single-cell proteomics workflows (SCP-W) and their associated sample preparation techniques.

## Single-cell proteomics workflow

The significant and game-changing innovations that led to the rise, maturation, and rapid march of single-cell proteomics and its various workflows are technical advancements in loss-less sample preparation techniques where the input material is a single cell. The field had initial success with large single cells like blastomeres [17] and oocytes. Virant-Klun et al. in 2016 published a prescient SCP study [18] where they used a novel sample preparation technique called single-pot solid-phase-enhanced sample preparation (SP3) using magnetic beads to investigate human oocyte biology and preimplantation development. The researchers consistently identified ~ 450 proteins from individual oocytes, which was a groundbreaking protein identification number at the time. Their effort paved the way for the forthcoming methods of quantitative proteomics at limited-cell and single-cell resolution.

### Our march toward a standardized SCP workflow

The community’s persistence and technical acuity have led to not only the identification but also the robust quantification of more than a thousand proteins for various types of cells. As seen in Table 1, the research community now has several SCP Workflows (SCP-Ws) to choose from and we highlight the various components of the current and most commonly used SCP-Ws. These include both labeled and label-free (LF) workflows. In this review, our main focus will be a review of the labeled SCP-Ws, since the Mechtler group has recently provided a comprehensive overview [19] of LF SCP-Ws, and we shall touch on these LF workflows only in a brief manner. What we want to stress in this review is the significant barrier to entry, in terms of expertise and expense, that an MS-based research lab faces if they are to initiate a program in SCP and that the whole effort that is needed may be quite daunting. This is unfortunate because in order to advance the field of single-cell biology research and deliver results that can be translated into clinical practice, we need many more MS labs participating in generating results and in the verification of novel findings by other labs. Sadly, at this point, only a few specialized labs have true access to SCP-based cutting-edge research. It can be made evident that the different SCP workflows vary quite a bit and are significantly nuanced. Therefore, MS labs around the world who *are uninitiated in SCP and do not have access* to the fairly deep expertise needed in cell sorting, specialized cell lysis techniques, labeled or non-labeled quantitative methods, sophisticated liquid chromatography (LC), mass spectrometry instrumentation, and advanced statistical analysis techniques do not have a fair shot at allowing access to scientists who want to utilize the power of SCP to answer deep biological questions that they seek to answer in all types of diseases.

**Table 1:** Primary components of current SCP-Ws

SCP workflow (SCP-W)	Cell sorting	Cell lysis	Labeling method	Labeling reagent	LC	LC active gradient (mins)	MS instrument	MS acquisition method	Protein coverage	Search engines	Throughput (SC/day)	Cost/cell	Inventor
SCoPE	FACS	mPOP	Isobaric	TMT/TMTPro	Nano LC	90	Orbitrap	DDA	1500–2500	Proteome Discoverer, Amanda, FragPipe	128	$	B. Budnik
SCoPE2	cellenOne	mPOP or nPOP	Isobaric	TMTPro	Nano-LC	60	Orbitrap	DDA	1500–2700	Proteome Discoverer, Amanda, FragPipe	384	$$$	N. Slavov
SCREEN	FACS	mPOP	Isobaric	TMT/TMTPro	Nano-LC	60	Orbitrap	Targetted	1500–4600	Proteome discoverer with Chimerys	384	$	B. Budnik
plexDIA	cellenOne	nPOP	Non isobaric	Non isobaric	Nano LC	30	Orbitrap	DIA	1500–3000	DIA-NN, MaxQuant, Spectranaut	144	$$$	N. Slavov
mDIA	cellenOne	Chemical	Label free	N/A	Evosep	31	timsTOF SCP	DIA	1500–4000	DIA-NN, MaxQuant, Spectranaut	160	$$$	M. Mann
One-Pot	cellenOne	Chemical	Label free	N/A	Nano-LC	20	Orbitrap	DIA	1500–2500	DIA-NN, MaxQuant, Spectranaut	72–188	$$	K. Mechtler
Nano-POTS	cellenOne	Chemical	Label free	N/A	Nano-LC	40	Orbitrap	DDA WWA	3000–3500	Proteome Discoverer with Chimerys	36	$$	R. Kelly

Most importantly, there is also a significant barrier to entry due to the upfront financial expenditure that is required to set up a successful single-cell proteomics laboratory. From our own experience, we can clearly state that this results in a misunderstanding of the technology and an unfortunate misuse of precious time and resources. Therefore, the specialized community of SCP researchers have an obligation to advance the field by coming up with a rigorous standard for SCP and its non-complex implementation that will help reduce the barrier to entry, thus allowing a larger number of MS-based research labs to participate fully and globally in this single-cell-based technological revolution.

### Taking guidance from the scRNA-seq field

Our task to standardize and simplify the SCP field need not be arduous or steeped in novelty because the single-cell revolution had started a while back with the advent of scRNA-seq technologies. As the field of scRNA-seq grew rapidly, it also generated a massive landscape of tools and analysis methodologies. A recent review [20] published in 2023 by Heumos et al. and the *Single-cell Best Practices Consortium* summarizes the available technologies and suggests comprehensive best practice workflows to help provide an entry point for novices in the scRNA-seq field and guidance to experts on current best practices. Therefore, we urge the SCP community to take this as an example to not only put together best practices but also borrow and adapt existing analysis tools and technologies that have helped the scRNA-seq field accelerate its mission.

### Data-dependent and data-independent acquisition methods

Before we go into the details of several SCP workflows, we highlight two recent studies to exemplify how far we have come since the studies by Virant-Klun and team. Vegvari et al. from Roman Zubarev’s group in 2022 demonstrated [21] that single-cell proteomics techniques are currently quantitative enough to study the drug effects on target proteins. This has ushered in single-cell chemical proteomics (SCCP) as a novel approach to explore the cells’ heterogeneous response to drugs. On average, they were able to identify and quantify over 1500 proteins and 10,000 peptides in single cells at each drug incubation time. But in order to make breakthrough scientific discoveries in drug discovery, we need to go much deeper into the proteome with techniques like SCCP. Echoing Zubarev, we also encourage the community to help achieve and then exceed the benchmark of 5000 proteins quantified with ≥ 2 peptides. As mentioned earlier, in 2023, Matzinger et al. from the Mechtler group published a detailed comparative study [19] in which they demonstrated that the data-independent acquisition (DIA) method is better than both data-dependent acquisition (DDA) and wide-window acquisition (WWA) methods and it achieved a proteome coverage of more than 2200 proteins from a single cell with a 20-min active gradient. An encouraging high-throughput label-free DIA method [22] has also been introduced in 2023 by the Parker group. They utilize a dual-trap single column (DTSC) and optimize it for nano flowrate (500 nL/min, NanoDTSC), which significantly increases the throughput of single-cell analysis. Using NanoDTSC, they were able to reproducibly quantify more than 1000 protein groups from individual cardiomyocytes over 15 min of total run time. Additionally, they demonstrated system robustness by running over 1700 samples without replacing any components. Now, the multiplexed labeling technique is used primarily in DDA and rarely in DIA, because present approaches generate more complex MS2 spectra, create severe ratio distortion, or lead to a reduction in quantification accuracy and precision. However, various SCP researchers have diligently explored coupling multiplexing techniques to DIA, for the obvious reason of increasing throughput as much as possible.

### The variety in labeling methods and interplay with SCP-W

With vastly improved techniques to robustly process a very tiny amount of sample from a single cell, while maintaining the fidelity of its inherent proteome and associated PTMs, coupled to advances in nanoflow liquid chromatography (LC), mass spectrometry (MS), and data analysis workflow, the field of SCP is emergent and quickly advancing the field of single-cell biology. As mentioned, researchers in this field have established a number of workflows to query the proteome of specific cells of interest.

Initial studies in SCP focused on the traditional label-free (LF) DDA methods, but soon, researchers started employing isobaric labeling like tandem mass tag^23^ (TMT; Thermo Fisher Scientific, Germany) and also non-isobaric labeling methods in order to respond to the need for higher throughput and better sensitivity. In this review, we will primarily focus on labeled workflows and associated sample preparation techniques available to researchers. For a comprehensive review [19] on label-free SCP (LF SCP) workflows, we recommend reading the recent work from the Mechtler group. In particular, label-free LF DIA is gaining traction in SCP and has already been used in a number of proteomics studies [24–28]. The well-established sample preparation techniques, reviewed below, are primarily incorporated into SCP workflows with DDA that utilize the most commonly used TMT isobaric labeling methodology. Although DIA is becoming increasingly popular, it is quite challenging to utilize it with isobaric labeling. However, a study [29] by Ctortecka et al. in 2022 provides a potential path forward with a DIA-TMT-driven SCP workflow. In their study using merged triplicates of HeLa and K562 mixes at 0.5-ng total peptide input, they demonstrate that the ID-independent DIA-TMT method has better reproducibility than the standard DDA methods. However, it remains to be seen how this method can be used in an SCP workflow.

We remind the readers that there are a number of labeling methods at our disposal. A common technique in quantitative mass spectrometry is to label the primary amines on peptides with reagents that contain stable isotopes. There are two main groups of labeling methods, namely *isobaric* and *non-isobaric*. The aforementioned TMT and iTRAQ [30] (isobaric tags for relative and absolute quantification) are isobaric, whereas mTRAQ (mass differential tags for relative and absolute quantification; SCIEX, Framingham, MA, USA), SILAC [31] (stable isotope labeling by amino acids in cell culture), and dimethyl labeling are non-isobaric methods. While there are pros and cons for each of these techniques, and selection of a technique should primarily be based on application, a study [32] published by the Carr group in 2012 demonstrates the superiority of the iTRAQ labeling technique over mTRAQ. The group reported iTRAQ labeling was able to quantify nearly three times more phosphopeptides and nearly twice as many proteins as mTRAQ. They concluded that iTRAQ has better sensitivity and reproducibility and exhibits less variability than mTRAQ-based quantification. However, as seen below, non-isobaric methods can be coupled with DIA to create an SCP workflow with encouraging results.

In this review, our focus will be on the TMT labeling approach. We highlight that labeling techniques have a rich history, and it is worth noting that the reductive demethylation derivatization was first described more than two decades ago in an original report by Hsu [33] et al. in 2003, and then expanded by Boersema [34] et al. and Taouatas [35] et al., from the Heck group. Recently the Mann group deposited a preprint report [36] in bioRxiv describing a novel non-isobaric SCP workflow involving multiplex-DIA (*mDIA*) where they used the dimethyl labeling technique on single-cell samples. As mentioned, non-isobaric methods have been around for a few decades, but since mass spectrometers are now much faster than a decade ago, SCP developers can take advantage of the increased MS speed and successfully add non-isobaric labeling to DIA. The field of neurobiology has seen the recent development of novel network-based complex data analysis methods. This now gives us the ability to identify the complex spectra that are generated by non-isobaric-labeled SCP methods. Therefore, improvements in both data analysis and instrumentation have brought about the emergence of *la*beled *DIA* (LADIA) methods which can potentially increase the proteome coverage in single-cell analysis, but this can only happen due to the original idea of the carrier channel that was proposed by one of us in the SCoPE-MS method [5]

The Mann group’s mDIA workflow was able to identify almost 4000 proteins for some of the cells, with a throughput of 80 single cells per day when using a reference channel. This is encouraging, though the throughput is at least a quarter less than that of the labeled method with DDA as demonstrated in our lab. So, in summary, the proteome coverage with LADIA will be on par with LF single-cell methods, but the throughput will be significantly higher LF SCP, e.g., LADIA will have a throughput that is up to 3–5 times higher than that of mDIA. What is really encouraging about the mDIA method is that due to the high sensitivity of DIA when using a reference channel, one can now perform analysis of single cells in a spatial context [37]. In 2022, Derks et al. published a method that used the mTRAQ labeling technique in another novel SCP workflow called plexDIA [38]. This workflow has been shown to reach a proteome coverage that is similar in depth to that of label-free DIA. When applied to single cells, *plexDIA* was able to quantify around 1000 proteins per cell with a ~ 5-min active chromatography per cell. Additionally, they were able to show that the quantitative accuracy and repeatability of plexDIA are similar to those of LF DIA. According to the researchers, plexDIA increases sample throughput by 3- to 12-fold over other best-of-class non-isobaric labeled single-cell proteomics methods [39, 40]. The authors conclude their report provocatively stating that they anticipate plexDIA to be the predominant DIA-driven SCP workflow and will be ranked higher in terms of preference than all other LF methods.

There is one other labeling method that would be useful to consider for SCP. In 2020, Tien et al. [41] presented an isobaric labeling technique based on the Ac-AG tag which has the advantages of reporter ion-based quantification of DDA but also allows DIA to be done in a multiplexed manner without sacrificing the data acquisition rate and complicating MS2 spectra. However, the main issue is that this method like plexDIA is inherently limited to low throughput since it currently can only be done in triplex.

### Labeled SCP workflow

Given the recent comprehensive review of LF SCP-W by the Mechtler group [19], here we briefly focus our attention on a few labeled SCP-Ws that we are most familiar with and are available to the SCP community and also discuss a novel workflow called SCREEN that we are developing in our Biomarker Discovery Laboratory (BDL) at the Wyss Institute.

As discussed earlier, initially, most SCP studies used a standard label-free DDA workflow. In order to increase sensitivity and get deeper into the proteome while increasing throughput, the SCP field started developing protocols that performed sample multiplexing with isobaric labeling strategies, like TMT. The first successful implementation of this strategy was published [5] by one of us in 2018 and was called *SCoPE-MS*, or *SCoPE* for short. A novel innovation in SCoPE was the introduction of the carrier channel, which allowed the field to move from quantifying 100 s of proteins to 1000 s of proteins per cell. By combining up to 18 samples with TMT18pro into a single sample, researchers were able to increase the protein coverage by up to a factor of 18. One of the TMT channels, called the carrier channel, is populated with up to 200 single cells, leaving one or two adjacent channels empty to avoid contamination leaking over from the carrier channel. By using the carrier channel, the mass spectrometer was now able to trigger more and richer fragmentation spectra resulting in a much greater number of peptide and protein identification, and simultaneously allowing relative quantification based on the TMT reporter ions. The innovation of multiplexing in conjunction with the carrier channel has been widely adopted [42, 43] with great success since the inception of SCoPE.

One other important innovation that allowed SCoPE-MS to be successful was the introduction of a novel technique for sample preparation. “*Minimal ProteOmic sample Preparation*” (mPOP) [44] was developed to reduce the losses that are incurred during sample cleanup when dealing with a very small sample amount from a single cell. It is a cell disruption technique and uses a physical rather than a chemical lysis protocol, and that is how mPOP gets rid of cleanup-related losses, since by design the cleanup step is totally avoided. This in turn simplifies the sample preparation process, thus making it faster and amenable to automation. The Slavov lab introduced *SCoPE2* [45] in 2021, which is a variation of the original SCoPE-MS workflow, of which Slavov and Budnik were co-inventors. The SCoPE2 workflow can use either FACS-sorted cells or a CellenONE robot to automate many of the steps, including cell sorting, and it is claimed that this allows the analysis of ~ 200 single cells per day. The authors state that the workflow can be fully automated using widely available commercial equipment, albeit some of these pieces of equipment come with a heavy price tag. The Slavov lab in 2022 also introduced a variation of mPOP called *“nano-ProteOmic sample Preparation”* (nPOP) [46], in which piezo acoustic dispensing is used to isolate individual cells in 300-pL volumes and all subsequent sample preparation steps are performed in small droplets on fluorocarbon-coated slides. Sample preparation, including cell lysis, digestion, and labeling of single cells, is performed in 20-nL volumes. In the SCoPE2 workflow, nPOP can also be used, but due to the nanoliter volume requirements, the commercially available cellenONE robot needs to be used and the process can be automated. This type of march toward fully automating the various SCP workflows is a very welcome sign for the field. We expect that ongoing development in this area, along with our own work, will very soon bring protein identification numbers to more than 5000 proteins per cell and SCP will become as equally important to single-cell biology research as scRNA-seq-based technologies.

It is well understood that the original and groundbreaking SCoPE-MS method has a fundamental limitation in the number of cells that can be analyzed, in addition to the number of proteins that can be detected across all single cells in a study. To address this and other inherent technical disadvantages of SCoPE-MS, a new method called SCREEN (Single Cell pRotEomE aNalysis) was introduced by one of us and reported in a preprint [47]. The stochastic nature of MS2 triggering that is inherent to mass spectrometers creates a problem in which peptides from many proteins do not undergo MS2 and this varies from cell to cell. Therefore, the data matrix that is produced includes a lot of missing values which we address in a non-ideal manner with various computational approaches. To address this problem of missing values and improve the situation, a new data acquisition method was proposed and that was to use a targeted method. The authors of SCREEN propose building a library of unique peptides drawn from the carrier channel before performing the real SCP analysis. This provides a significant advantage by allowing the instrument to trigger only on peptides that have been pre-specified within a certain chromatographic time window. Therefore, this method provides a much more comprehensive and deeper proteome coverage by targeting low-abundant peptides. Additionally, it helps alleviate the missing value problem by having the same peptides triggered and quantified across many MS runs. With SCREEN, the authors provide a high-throughput, robust, fast SCP workflow that has increased protein identification and protein quantitation ability that spans across more cells than previously achieved. They applied this workflow to analyze the single-cell proteomic landscape of cancer cells under drug response to uncover heterogeneity in cellular response, which appears to be the first of its kind. The SCREEN workflow uses FACS for cell sorting, an automated mPOP method for cell lysis, digestion, and TMTPro labeling, followed by LC–MS/MS on an Orbitrap using DDA and an active 60-min gradient to identify ~ 4600 proteins with a Proteome Discover::Chimerys search engine, which to our knowledge is one of the largest number of proteins ever identified by an SCP workflow. We continue to improve SCREEN in our lab and plan to cross the 5000-protein identification number very soon.

### Additional sample preparation techniques

In addition to the two sample preparation techniques already described, namely mPOP and nPOP, there are two other techniques that need mentioning. It is appreciated that if we move from macroscale sample preparation techniques to micro- and nanoscales, then we have a better chance to profile a small number of cells, and single cells, more deeply and likely to be relevant in real clinical settings. In 2018, the Kelly group published [48] the first of its kind nanoscale sample processing technology for mass spectrometry–based proteomics workflows. This was called *“Nanodroplet Processing in One pot for Trace Samples”* (*nanoPOTS* or *nPOTS*). In addition to using nanoPOTS in the single-cell proteomics workflow, the authors propose that it can potentially be used for tissue imaging at the proteome level. At that time, nanoPOTS was limited to as few as 10 cells, but recently, in 2022, the Kelly group submitted a preprint [49] in bioRxiv where they reported combining nanoPOTS sample preparation and ultra-low-flow LC with the newly developed wide window acquisition (WWA) to quantify more than 3000 proteins from single-cell fast label-free analyses. These are encouraging results, and we look forward to continued improvements to this workflow.

In 2021, Weke et al. published [50] a recently developed microscale proteomic method called *“Microdroplet Processing in One pot for Trace Samples”* (microPOTS or mPOTS), which they used to identify proteomic changes in ~ 200 Barrett’s esophageal cells after physiologic and radiation stress exposure. This technology was an adaptation of nanoPOTS, uses conventional micropipettes, and operates in a low-microliter range. This was developed to specifically tackle a few bottlenecks such as the demands for nanoliter pipetting and specialists to run nanoPOTS. Woo et al. recently reported [51] in 2022 the detection of proteome changes in macrophage activation and classified human lung cells into distinct populations. Here, they use the microPOTS technology for MS library generation with 50 sorted cells and sort single cells into nanoPOTS chips for MS analysis. This is an example of how both the micro- and nanoPOTS chips were used in an SCP workflow.

The selection of specific analytical instruments, e.g., mass spectrometers, LCs, etc., and labeling techniques that are used in a given SCP workflow has the potential to impact the quantity and quality of single-cell proteomics data. As far as we know, no rigorous study has been conducted to compare and contrast the implication of instrument choice on various SCP workflow outputs. TOF instruments are primarily used for label-free workflow, while Orbitraps are used for labeled workflow. In the following section, we highlight the work by the Orsburn group where they show very good results [55] when combining labeling techniques with a TOF mass spectrometer. Without a direct comparison of the same study on an Orbitrap, it is difficult to properly compare and contrast labeled techniques on a TOF versus an Orbitrap, though similar studies on an Orbitrap had better precision and reproducibility. On the other hand, the Mechtler group recently demonstrated [52] that a label-free workflow with an Orbitrap provides good precision and reproducibility, which leads us to conclude that the SCP workflow with an Orbitrap may have better precision and reproducibility than a workflow with a TOF-type mass spectrometer. It should be mentioned that there is also a strong push toward a workflow that utilizes ultra-short 5-min gradients in DIA mode. Suffice to say that the jury is still out on the topic of best SCP workflow and associated instrumentation as the field continues to vigorously innovate and improve the technology.

The need to develop a standardized, robust, scalable, and high-throughput SCP workflow.

As seen from Table 1, if we focus on just two components of the workflow, then the different labeling methods can be combined with various sample preparation techniques in either a DDA or DIA mode to create a specific SCP workflow (SCP-W). Many such combinations exist and others are being explored. Depending on the SCP-W employed, one immediately can appreciate the arrays of results that researchers can achieve to answer a given deep biological question. In order for SCP-W-driven novel discoveries to be translated into a clinical setting, we must first generate robust results that can be easily reproduced by others. Our work with SCP at the Biomarker Discovery Lab at the Wyss Institute, Harvard, is driven by unmet clinical needs in the drug target and biomarker discovery space. We are in the process of developing a SCP-W based on SCREEN that is robust, high throughput, and scalable so that it can be easily implemented in labs around the world. The development of an easily implementable SCP-W will allow researchers to engage in novel discoveries knowing that others can comfortably reproduce the results, thus increasing the likelihood of translation, which ultimately is the goal of applied biology.

## Biological applications

We highlight three recent biological applications where single-cell proteomics was instrumental in elucidating key insights which otherwise would not have been possible by means of traditional bulk sample-based proteomics investigations. A recent article [53] in 2023 by Mansuri et al. provides a mini review of the advances in single-cell proteomics and its application in uncovering hidden truths in biology. It provides a brief summary of the field and is a nice segue to the following three examples showcasing SCP applications in biology.

Schoof et al. in 2021 published a study [54] using the SCoPE workflow to characterize cellular hierarchies. To our knowledge, this was the first-of-a-kind study where SCoPE was used to perform single-cell analysis of leukemia hierarchy. The researchers were able to convincingly show that within their model acute myeloid leukemia (AML) system, the FACS data was recapitulated by the SCP data. They used a standard EASY-Spray trap column LC setup with relatively low flow (100 nl/min) and a 3-h LC method, coupled to an Orbitrap Exploris™ 480 MS with gas-phase fractionation provided by the FAIMS Pro instrument interface. With this setup at a throughput of 112 cells per day, with 14 cells analyzed per cell, they are able to routinely quantify ~ 1000 proteins per cell across thousands of individual cells.

Soon after the Schoof et al. publication, Orsburn et al. also in 2021 reported a study [55] showing SCP as a powerful tool to understand cellular processes at single-cell resolution. The researchers present a first in class application of SCP to the study of drug mechanism in single cells by treating a model KRAS^G12C^ mutant cell line with the covalent inhibitor sotorasib. They used the SCoPE2 workflow with a trapped ion mobility time-of-flight (TOF) mass spectrometer (timsTOF) to acquire over 40,000 tandem mass spectra in 30 min. Using the KRAS model human-derived cell line, they were able to quantify over 1200 proteins per cell. Additionally, to our knowledge, for the first time, Orsburn et al. successfully demonstrated the detection of multiple classes of post-translational modifications (PTMs) in single cells. They report over 2000 high-confidence peptide spectral matches (PSMs) for sequences containing the following modifications: phosphorylation, acetylation, methylation, dimethylation, succinylation, hydroxybutylation, crotonylation, and cysteine trioxidation. This indeed is a significant achievement for the SCP field as it opens up an extremely rich avenue for single-cell PTM (scPTM) research. Interestingly, they also point out that the primary limiting factor in SCP appeared to be the total concentration of each protein in a single cell, irrespective of the hardware configuration used. This study is an excellent example of the power of SCP in pharmacological studies.

Another study we would like to highlight is the 2022 bioRxiv preprint work [56] of Rosenberger et al., from the Mann group, where they describe a novel technology called single-cell deep visual proteomics (scDVP) and how it was used to resolve the context-dependent, spatial proteome of murine hepatocytes from a slice of a cell. This is a significant achievement for the SCP field as the researchers were able to successfully combine microscopic imaging data with ultra-high-sensitivity proteomics. They achieved this feat by building on four crucial technological advances, namely AI-assisted segmentation and laser microdissection, mDIA, low-flow gradients, and the ultra-high sensitivity of a timsTOF SCP mass spectrometer. With their scDVP workflow, the researchers were able to get more than 1700–2700 proteins per single cell shape, despite the fact that the sections had been fixed, stained, imaged, and laser dissected. They expect to enhance the coverage depth since the primary limiting factor is MS sensitivity, which we expect will continue to improve. There are many more past and current studies that we can highlight, but hopefully, these provide the readers with examples showing the true and continuously improving power of SCP technologies.

As a final example, we provide a flavor of multi-omic workflow [57] where single-cell (phospho-)protein and RNA detection was integrated to discover high-resolution phenotypic characteristics and active signal transduction of human antibody-secreting cells (ASCs). This study at the single-cell level, mostly likely for the first time, mapped phenotypic markers and recorded active signal transduction in a heterogeneous mixture of differentiating and antibody-secreting B cells. The authors expect that this novel strategy would help further the understanding of human ASCs in healthy and diseased samples in order to identify novel biomarkers and drug targets.

To summarize, we would like to stress that the deep biological insights achieved in the above examples would not have been possible with traditional bulk proteomics methods, but only with single-cell proteomics workflows. SCP is still a young but accelerating field in the arena of single-cell biology, and very soon, we expect to see important discoveries from SCP leading to positive outcomes for patients.

## Conclusions and future perspectives

The field of single-cell proteomics is in its fifth year after the publication of the SCoPE workflow^5^ and since its publication the field has exploded with additional innovations and applications. The state of SCP innovation and development is very encouraging for the field of single-cell biology as it continues to mature and evolve. However, access to what SCP can offer is limited to a handful of specialized academic groups with very limited expansion into industry. The primary bottleneck to wider adoption is the lack of a robust, automated, and accessible sample preparation platform. Currently, there are only two commercial platforms that provide needed sample preparation solutions, which are fully automated and include cell sorting. The dominant platform is CellenONE (Cellenion; Lyon, France). Hewlett-Packard (HP; Palo Alto, CA, USA) also provides a semi-automated platform, D100 Single Cell Dispenser, that has recently been introduced onto the market. We are aware of several other companies in the process of developing their own solutions, but we urge that expert SCP research labs work closely with industrial partners so that the solution is purposefully built and delivered. For ubiquitous acceptance of single-cell sample preparation platforms, we need more industry and academic collaborations producing robust, industrial strength, easily accessible, appropriately priced products. Industry has already demonstrated that mass spectrometry can deal with low protein amounts from single cells, and these technologies have been widely accepted.

The next big challenge for SCP is the standardization of scientific studies, so that the field can produce results that are robust, reproducible, and verifiable by others. In order to achieve this, we need to build the foundation for standardization on two primary pillars: standards that can be run to qualify the sample preparation workflow and standards that can be used to qualify the instrument and chromatography capabilities of the various systems that are represented in analytical labs worldwide. Also of importance are the standards pertaining to the analysis workflow. This at a minimum needs to be inter-exchangeable between different SCP-Ws, so that the results can be verified independent of the systems used to produce them. In other words, the biological truths should be the same irrespective of the systems and methods used.

When we look at what has happened in the field of scRNA-seq, we notice that more than 1000 different data analysis packages have been developed over the last decade. We need to learn from our colleagues in the transcriptomic and genomic fields, so that we may earnestly avoid that type of chaos. Without standards, without checks and balances, the multitude of investigators deploying countless unverified analysis methodologies can produce erroneous conclusions based on inaccurate mathematical approaches. As a result, the whole field of biology suffers in the short and long run, which then does a disservice to those who we ultimately want to help, i.e., patients.

Now, given the experiences and lessons in transcriptomics and genomics, we have an advantage with SCP if we can adequately learn, adopt, and adapt. Understanding cell heterogeneity is critical to holistic and accurate knowledge generation in all fields of biology. Follow-on clinical applications are fraught with errors if we do not properly employ SCP to understand cell heterogeneity. Areas of biology where cell heterogeneity is involved, but not limited to, are cell cycle studies, biology of disease, drug resistance, cell to cell interactions, and epigenetics. Since correlation between genes and proteins, and transcripts and proteins have proven to be very low, both areas now require a new wave of analysis to be performed at the protein level. That is the only way we can truly understand the mechanisms of the complex biological machinery within a cell. Thus, single-cell proteomics has a bright future ahead of it.

In summary, we envisage a very positive outlook for the field of single-cell proteomics. We urge the SCP community to band together, accept the lessons learned from the single-cell genomics and transcriptomics field, set standards so as to unify our collective efforts, and hold one another accountable where the fight for biological truth is the only currency. We hope that very soon, with rigorous standards in place, we will be able to couple SCP to scRNA-seq and subsequently to other single-cell-based -omics approaches and launch a revolution in single-cell-omics (SCOmics) so that we can holistically answer single-cell biology questions and solve truly meaningful problems in health and disease.
